# Implementing Peer Support in Community Mental Health Nursing Teams: Qualitative Evaluation

**DOI:** 10.1111/jpm.70076

**Published:** 2025-12-12

**Authors:** Nora Ambord, Christian Burr, Sabine Rühle Andersson, Anna Hegedüs

**Affiliations:** ^1^ Department of Health Professions Bern University of Applied Sciences Bern Switzerland; ^2^ University Hospital of Psychiatry and Psychotherapy, Bern University Hospital for Mental Health Bern Switzerland; ^3^ RG Mental Health Nursing Central Institute of Mental Health Mannheim Germany

**Keywords:** community mental health nursing, implementation science, mental health recovery, mental health services, participatory research, peer support

## Abstract

**Introduction:**

Peer support (PS) is considered central in the recovery for people with mental health problems. While PS is established in the inpatient setting in Switzerland, it is rarely available in outpatient settings.

**Aim:**

To evaluate the implementation of PS within Swiss community mental health nursing (CMHN) teams, focusing on its content and impact, as well as the factors contributing to its successful implementation and the potential barriers.

**Methods:**

We conducted a qualitative, participatory study using implementation research principles in three CMHN teams. Data collection included interviews with managers, peer support workers (PSWs) and nurses, along with participant observations. Findings were analysed using qualitative content analysis. This article follows SRQR guidelines.

**Results:**

Clients and staff reported high satisfaction with PSWs but PS was only implemented at the client level. Challenges included unclear job descriptions, low PSW working hours, limited understanding of PS and financial constraints.

**Discussion:**

There was a lack of adequate training on PS and recovery. PSW were seen somewhat externally by the CMHN teams which weakened integration.

**Conclusion:**

Aligning with best practice guidelines, our findings suggest that successful implementation of PS requires standardised PSW training, financial recognition, comprehensive team training and internal efforts on PSW team integration.

**Relevance Statement:**

This study provides practice‐based insights into implementing peer support in Swiss community mental health nursing teams, a newly emerging area of practice. It highlights key factors influencing peer support worker integration, including role clarity, training gaps and nursing team preparation. The findings offer guidance for community mental health nurses to strengthen recovery‐oriented care through the effective implementation of peer support. By addressing practical barriers and facilitators, this study directly supports the advancement of mental health nursing in community settings.

## Accessible Summary

- What is known on the subject
  ○ Peer support is crucial for mental health recovery, but in Switzerland, it is mostly used in inpatient settings.
  ○ In outpatient settings, and especially in community mental health nursing teams, peer support is not common.
- Originality
  ○ This study explores how peer support workers can be successfully integrated into community mental health nursing teams.
  ○ It examines the role and perception of peer support work by clients and nursing teams.
  ○ The study uses a participatory approach, involving service users at every stage of the research.
- Significance
  ○ Implementation strategies were adopted based on organisational needs and knowledge.
  ○ Challenges include low employment levels, billing issues within the Swiss health insurance system and limited knowledge on recovery principles.
  ○ Nursing teams generally welcomed peer support and saw positive effects on clients and high levels of client satisfaction with outreach peer support were noted.

## 1 Introduction

Peer support (PS) is a central element in the recovery process of people with mental health problems. Guidelines (DGPPN—Deutsche Gesellschaft für Psychiatrie und Psychotherapie 2019; World Health Organization (WHO) 2021) recommend its integration into psychiatric services as part of person‐centered and human rights‐based mental health approaches. Peer support workers (PSWs) are individuals with personal experience of mental health problems and service use, who use their insights and skills to support others with similar conditions (Davidson et al. 2006). PSWs facilitate, guide and mentor others by fostering hope, demonstrating recovery in action and empowering individuals to rebuild fulfilling and meaningful lives, connected to communities and social circles of their choice (Stratford et al. 2019). Research on the effectiveness of PS remains limited, though studies have demonstrated positive impacts on outcomes such as hope, quality of life, personal recovery and empowerment (Chinman et al. 2014; Cooper et al. 2024; Fuhr et al. 2014; Yim et al. 2023). While the effects on clinical, personal and functional recovery are more evident for individuals with serious mental illness, PS is considered to have potential benefits across various mental health conditions and treatment approaches (Smit et al. 2022).

PS is increasingly being introduced into mental health services internationally in various settings including inpatient, outpatient, digital and community‐based services and is often delivered in one‐to‐one or group sessions (Fortuna et al. 2022; Gillard et al. 2017). In Switzerland, PS has developed steadily over the past decade in mental health care, especially in the German‐speaking regions (Burr et al. 2022). One key driving factor was the PSW training program ‘Experienced‐Involvement’ (EX‐IN), which was introduced in 2010. The program consists of ten three‐day sessions spread over 1.5 years and has been offered approximately once per year since its inception (Hegedüs et al. 2021). A survey from 2017 including 125 PSWs in Switzerland revealed that most worked directly with psychiatric service users or in education and were employed in inpatient settings and often in part‐time roles (Burr et al. 2020). The limited presence of PS in outpatient settings contrasts with the existing evidence highlighting PSWs' crucial role in the community setting in addressing disparities in access to psychosocial care for marginalised groups (Gillard 2019). This gap is further emphasised by the evaluation report on the implementation of the Convention on the Rights of Persons with Disabilities (2022) in Switzerland, which identified a significant lack of community‐based, preference‐driven psychosocial services and support.

Community mental health care in Switzerland is, among others, provided by home care services which are available across the country and provide a wide range of nursing services, including specialised community mental health nursing (CMHN) (Stocker et al. 2016). These CMHN teams are well positioned to fill the aforementioned gap because they are available across Switzerland and because PSW has already been implemented in some organisations. Since these teams are not organised on an interprofessional basis, unlike in international contexts, there is little knowledge about the specific challenges and requirements for implementing PS in these settings.

PS was implemented within the first CMHN teams in 2020. The driving force behind this initiative is the INGA project, which aims to support CMHN teams in the implementation of PS roles. It offers fee‐based services, including PSW recruitment, team training and the integration of PSW into regular services, along with ongoing development and support of PSWs in these services. Research (Ibrahim et al. 2020; Mancini 2018) has highlighted the importance of organisational culture and staff attitudes, emphasising that these elements must be carefully considered for effective integration. The current challenge in Switzerland is that the implementation of PSWs in CMHN teams is occurring in an area with limited experience and knowledge of PS, where a basic understanding has yet to be developed and funding for PSWs remains insufficiently regulated. Nevertheless, there is considerable willingness and growing recognition of the potential for PS to improve practices and services for users in psychiatric home care services. Considering these aspects, it is essential to assess PS implementation in this specific mental health service setting. The aim of this study is therefore to evaluate the implementation of PS within Swiss CMHN teams, focusing on its content and impact, as well as the factors contributing to its sustainable and successful implementation and the potential barriers.

## 2 Methods

### 2.1 Design and Theoretical Framework

In this retrospective cross‐sectional study, we used a qualitative, participative, content analytic approach to evaluate the implementation of PS in CMHN teams. This approach aligns primarily with phenomenological‐hermeneutic and social constructivist traditions, aiming to systematically capture subjective meanings and shared patterns within textual data (Creswell and Poth 2016). A researcher with lived experience as a psychiatric service user was part of the team and involved in all stages of the research process. Our research team has expertise in mental health nursing science, practical psychiatric nursing experience and specialised knowledge in recovery‐oriented care, with a focus on integrating PSW involvement in clinical practice and research. The evaluation was guided by the Consolidated Framework for Implementation Research (CFIR) (Damschroder et al. 2022). The CFIR is a comprehensive and widely used framework in implementation science that helps identify and address key factors that may influence the implementation of evidence‐based interventions. It supports the evaluation of implementation strategies, context analysis and the identification of implementation barriers. Its structure comprises 5 domains. To address the aim of assessing the contents and impact of PS in the outpatient setting, this article focuses on the *Innovation* and *Inner Setting Domain* of the framework. The *Innovation Domain* focuses on ‘the innovation’ being implemented; in our case, this is PS. It includes aspects such as source, evidence, advantage, adaptability, trialability, design and costs. The *Inner Setting Domain* refers to the environment where the innovation is implemented; in our case this is the CMHN team. Sub‐categories are structure, relationships, communication, culture, change readiness, priority, incentives, mission alignment and resources. The present article was written in accordance with the Standards for Reporting Qualitative Research (SRQR) (O'Brien et al. 2014).

### 2.2 Context and Setting

We evaluated the implementation of PS in three CMHN teams of home care services in the German‐speaking part of Switzerland. Using purposive sampling, we included organisations at different stages of PSW implementation. At the time of data collection, one organisation had introduced PS only a few months earlier, another about a year prior and the third had implemented it 3 years earlier. This variation allowed us to examine the entire implementation process through cross‐sectional interviews. During implementation and ongoing development, all three organisations were supported by the services of the INGA project.

Community mental health nursing in Switzerland is financed by the health insurances on a time basis. To bill these costs, individuals must at least have training as nursing assistants. This also applies to PSWs working in home care services. This training focuses on somatic care and lacks alignment with the psychiatric competencies central to their role.

### 2.3 Data Collection

Data collection was conducted between November 2023 and February 2024 and encompassed the following steps: (1) a focus group interview with members of the INGA project, (2) expert interviews with PSW, nurses supervising the PSW and managers in the CMHN teams, (3) participatory observations of peer‐client visits and (4) a stakeholder workshop.

The interview guide for the focus group interview included questions on the intervention, external influencing factors and the implementation process (e.g., training, coaching, supervision). The guides for the expert interviews were developed in accordance with the CFIR (Damschroder et al. 2022), with slight adaptations tailored to each of the three professional groups. Management interviews focused on external factors and the implementation process, while interviews with peers and nursing staff addressed the intervention, internal organisational factors and staff characteristics. The participant observations and peer interviews were conducted by a researcher with lived experience as a psychiatric service user. This participative approach fosters a more authentic and trusting rapport with participants, while minimising disruptions to the natural dynamics of peer‐client interactions (Patterson et al. 2014). The focus group and expert interviews were recorded on‐site using audio files, while the participatory observations were documented with site notes by the researcher.

### 2.4 Data Analysis

All interviews were transcribed from Swiss German dialect into High German using the rules of Dresing and Pehl (2018). All text files including the site notes of the participatory observations were analysed using qualitative content analysis, following a mixed deductive and inductive approach as described by Elo and Kyngäs (2008) and Saldaña (2013). The domains of the CFIR served as the theoretical framework for defining the main categories. The development and allocation of codes within the three categories were carried out inductively. The CFIR framework is designed to describe facilitators and barriers to implementation outcomes (Damschroder et al. 2022). Accordingly, we systematically identified these factors within each domain and synthesised them for the purpose of this publication. Preliminary findings were discussed and validated in a member check with PSWs as well as in a stakeholder workshop with professionals working in various psychiatric home care organisations, PSWs, experts on PS, caregivers as well as other interested people. Given the central role of PSW in this study and their current status as a minority group in healthcare, it was crucial to ensure their perspectives were accurately represented. The feedback provided was carefully incorporated into the analysis to enhance the accuracy and depth of the findings as part of an iterative approach. The transcription and analysis were carried out using MAXQDA 24 (VERBI Software 2024).

### 2.5 Ethics

The ethics committee of the canton of Berne concluded that ethical approval was not required for this study (Req‐2023‐01242) as it is not subject to the Human Research Act in Switzerland.

All participants received detailed study information, including their right to withdraw at any time. They provided written consent, confirming voluntary participation, and were given a copy of the consent form.

## 3 Results

Between November 2023 and February 2024, one focus group interview, 9 expert interviews and 3 participatory observations were conducted including a total of 18 participants. At the end of the data analysis, in June 2024, a stakeholder workshop with 16 participants was carried out. Further information on the sample is given in Table 1.

**TABLE 1:** Data collection methods and sample sizes.

Data collection method	Participants	Number of participants (*n*)
Focus group interview	Members of the INGA project	3
Expert interviews	A PSW, a nurse and the manager from each of the three organisations	9
Participatory observations	One PSW and their client in each organization	6
Stakeholder workshop	Nurses and managers working in psychiatric home care organisations, PSWs, PSW trainers, caregivers, members of INGA	16

The results are organised based on the themes in the CFIR domains *Innovation* and *Inner Setting* (Table 2).

**TABLE 2:** CFIR key themes allocation.

**Innovation domain**
Requirements for PSW
PSW‐client allocation and PS discontinuation
Description of PSWs' daily tasks
Impact of PS
Funding and Financial Dynamics of PS in Swiss Outpatient Care
**Inner setting domain**
CMHN team preparation
Process of PSW integration

### 3.1 Innovation Domain

#### 3.1.1 Requirements for PSW

Some requirements for PSWs working in the outpatient setting were highlighted. It was emphasised that PSWs working in CMHN teams must have a high degree of independence, as they often work alone. Participants from all three groups (management, nursing staff and PSW) stressed the importance of mental stability as a prerequisite, given that PSWs work autonomously, like the following quote from one of the nurses illustrates:

And, um, what I think is a big difference compared to the inpatient sector is that they (PSWs) are on their own (with clients). I think that requires even more, to be able to keep both feet on the ground and also (…) the expectation that they will get help when things get difficult. (…) We need to do that too. (Interview Nurse)

The educational background of the PSWs was heterogeneous. Only one of the three PSWs had completed the EX‐IN training. However, all PSWs had completed the required nursing assistant course. In this context, nurses and managers expressed concerns about the PSWs' limited psychiatric expertise.

#### 3.1.2 PSW‐Client Allocation and PS Discontinuation

In all three organisations, PSWs and nurses closely collaborated in client care. Nurses often decided which clients received PS, as PSWs worked part‐time (1–2 days per week) and did not visit all clients. Client selection was described as challenging; some nurses matched the PSW's background with the client's diagnosis, while others did not. After selection, an initial meeting with the nurse, PSW, and client was held, and both the client and PSW decided whether to continue with PS.

So as I know it now, there is always a getting to know each other first and I as a PSW can then say first, no, I can't imagine that. The client can also say, no, it doesn't make sense, or I don't like it, or whatever. (Interview PSW)

PSWs discontinued support for various reasons, including changes in the client's condition or the client no longer needing CMHN services. One PSW emphasised the importance of a proper farewell, explaining that she would call a client to say goodbye if a face‐to‐face meeting was not possible.

#### 3.1.3 Description of PSWs' Daily Tasks

A common approach involved PSWs and nurses alternating client visits every 2 weeks. PSW‐client visits took place in various settings, including clients' homes, outdoors and occasionally in cafés. One PSW frequently initiated outdoor meetings, incorporating activities like Nordic walking. PSWs often began visits by asking about the client's well‐being and discussing their immediate needs, adopting a situational approach rather than working with set goals.

Just be there and see what is needed. What is needed today. Do they need to build up their activity today, or do they need to talk today, or have we reached the point where we can tackle things? Like tidying up an apartment or doing something that the clients haven't got around to doing otherwise. That's why I said, first of all, to go in without a plan and then during this time, i.e., during the five to ten minutes when I feel what it could be today, to then implement it. (Interview PSW)

Tools such as games, apps, workbooks and social‐psychiatric materials varied depending on the PSW. PS included assistance with housing and job searches, promoting healthy behaviours, reducing compulsions, assistance with phone calls, fostering hope, active listening, support with self‐care, providing information on local support services or completing ‘homework’ from psychotherapy together. Job descriptions that clearly defined the tasks of the PSWs did not exist. Two of the three PSWs reported having considerable freedom in their work with clients, while one expressed a desire for more autonomy. Collaboration with nurses occurred intermittently, with joint discussions on the further course of action for client care. In this regard, a nurse emphasised that PSWs were required to write progress reports.

The findings revealed that PS was primarily viewed as an isolated measure focusing on PSW‐client interactions. Only two of the nine respondents mentioned that part of the PSWs' role involved offering a new perspective on challenges and contributing their suggestions and inputs to the other professionals in the CMHN teams. PSWs were mainly expected to adapt to the existing system, rather than being viewed as an opportunity to rethink or innovate within the whole organization.

We want to incorporate this knowledge through experience of the PSW. Because he has experienced this with a self‐awareness, as a complementary measure to our outpatient psychiatric home nursing. (Interview Nurse)

#### 3.1.4 Impact of PS

None of the psychiatric home care organisations had systematically evaluated the impact of PS or client satisfaction. However, several interviewees mentioned receiving informal feedback from clients, who reported a high level of satisfaction. During participatory observations, clients expressed feeling understood, hopeful and empowered by their interactions with PSWs, which they perceived as being based on mutual respect and equality. PSWs' experiential knowledge was highlighted as a key component of the support, fostering a deeper connection in the relationship with clients, as described by one of the managers:

Where (peer support) can supplement the treatment setting, where it can perhaps create a different openness, a different sense of being affected, a different approach, um, it can perhaps influence the recovery process. (Interview Manager)

#### 3.1.5 Funding and Financial Dynamics of PS

The PSW expressed satisfaction with their salaries, although concerns were raised about the comparison between PSWs' salaries and those of other healthcare professionals who have undergone several years of education.

Of course, I don't know what triggers it in my colleagues. Then maybe one of them would say, well, then I should get depressed, then I can register as a PSW and then I'll earn more than I would in three years of work and basic training. (Interview Manager)

As the mandatory nursing assistant training for PSWs focuses solely on somatic care without tackling PS and psychiatric competencies, interviewees expressed frustration and dissatisfaction with this training.

So we did the training and it was more in the somatic field. And then I did an internship in the somatic field. I think that it was billing‐related. That was very questionable for me. Because I'm not going to go and wash my clients, etc. (…) I saw a lot of things and learned new things. I don't mind new things. But it's totally different from what I need here. (Interview PSW)

Furthermore, the issue was raised that the additional costs for supporting PSWs and implementing the program with the help of the INGA project complicate the financial sustainability. These costs cannot be covered by the revenue generated from the PSW services billed through health insurance. Stakeholder workshop participants emphasised the need for greater advocacy to address these issues on a system level.

### 3.2 Inner Setting Domain

#### 3.2.1 CMHN Team Preparation

Preparation processes supported by INGA differed across the three teams. In one organization, a project group was formed. All teams received information on PS, provided either by an INGA representative or team manager. INGA also shared informational documents, though not all interviewees seemed familiar with them. Responses suggested the training focused mostly on providing information.

We received a lot of information, but no further training. (Interview Nurse)

One team declined a specific training on recovery and PS, as they were, from their point of view, already familiar with those topics.

#### 3.2.2 Process of PSW Integration

Nurses and managers reported that during the early stages of integration, team members expressed concerns about the resilience of PSWs and their capacity to handle the demands of working within the CMHN teams. Factors facilitating team acceptance of the PSWs were the involvement of team members who were already familiar with PS, clarity in the administrative processes, and the successful implementation in other home care organisations that served as a model.

There are people in the team who are already familiar with it (peer support) and think “wow, great, let's do it”. They then also inspire others. (Interview Nurse)

A PSW noted that, even after starting their work, some colleagues within the organization still lacked a clear understanding of what PS entails. He/she described the organisational climate as ‘chronically stressed’ with little effort made toward PSW integration. Additionally, the PSW reported missing a clear point of contact and experiencing discrimination due to personal mental health history.

Well, I have also heard someone saying, I don't know now whether (name of PSW) is allowed in a room where there are medications. Where I then have to say, firstly, this is discrimination and secondly, um, I don't have a problem, I've never had a drug abuse. This makes me feel attacked. Just because I'm a PSW. (Interview PSW)

This stood in contrast to the experiences of other PSWs, who described their encounters with the organisational culture as positive, highlighting its flat hierarchy and openness toward PS. A nurse emphasised the active role of CMHN managers in PS integration, viewing their strong involvement as beneficial for supporting the integration process. A PSW mentioned feeling valued as a full team member, appreciating the openness to their inputs and the high level of understanding.

You really get involved. As a newcomer, you are directly integrated into the team and supported by everyone. (…) So it's an open culture. (Interview PSW)

Across all three organisations, there was also some ambivalence regarding the equal treatment of PSWs. While some emphasised the importance of treating PSWs the same as other employees, specific examples revealed that this was not always reflected in practice. This was notably the case in situations where PSWs were meant to be shielded from overburdening and therefore were treated differently.

I would say that you just can't ask too much of the PSW. If it's a situation that's a bit difficult professionally, or something like that, I think that's almost the most important thing. That you don't leave them alone. (Interview Nurse)

Some nurses and managers reported that integrating PSWs into the nursing teams was challenging, primarily due to the PSWs limited working hours. As a result, they were often absent from joint lunches or team meetings. The interviews also suggested that the absence of PSWs from team meetings was partly due to the PSW exchange forums (equivalent to team meetings) offered by INGA, which were held externally and thus separate from the organization.

It's mainly at the team level. Exchanges are really difficult and it's difficult to get to know them (PSW). For some, they are still a bit out of place to us and I don't know how to solve this with the low working hours. (Interview Manager)

### 3.3 Facilitators and Barriers to the Implementation of PSWs in CMHN Teams

Table 3 summarises the factors that acted as facilitators or barriers to the successful implementation of PSWs into the CMHN teams. The findings indicate that whether PSWs had been part of the CMHN team for only a few months or several years was less relevant for successful integration than other factors identified.

**TABLE 3:** Factors contributing to PSW implementation in CMHN teams.

**Facilitators**
Other CMHN teams acting as models for successful implementation.
Team members as opinion leaders who are already familiar with PS and help drive its integration within the organization.
A recovery‐oriented approach within the team.
A team culture that is open to new ideas and change.
Flat hierarchies and an accessible leadership structure.
Managers with a positive attitude toward PS and close involvement in the implementation process (e.g., participation in working groups).
The presence of a clear contact person for the PSW within the team.
External support (such as INGA) during the initial implementation phase for: PSW recruitment, provision of training materials, adaptation to organisational context, etc.
**Barriers**
Unclear or nonexistent job descriptions.
Inadequate billing system through health insurance for PS in CMHN.
A ‘chronically stressed’ and hierarchical climate within the organization.
Lack of knowledge about recovery and PS within the team.
Low working hours of PSWs.
Non‐participation of PSWs in team meetings (e.g., due to autonomous offers from external support organization).

## 4 Discussion

The results showed that satisfaction with PS was generally high among clients, managers and nurses, indicating a positive impact of its implementation. While the organisations adopted different implementation strategies, common challenges emerged regarding PSW integration. These challenges mainly stemmed from the low employment rate, billing difficulties of the Swiss health insurance system, unclear role definition, variations in prior PSW training and inconsistent team training resulting in low understanding of key concepts such as recovery.

Our findings on barriers and facilitators for successful implementation of PSW within Swiss CMHN teams align with those of international literature from diverse settings, suggesting that the setting itself may not be a decisive factor. Previously identified relevant facilitators include clear role definition, training of PSWs and non‐PSW staff as well as staff attitude and the organisations' culture (Ibrahim et al. 2020; Mutschler et al. 2022), all of which we also identified as significant contributors in our evaluation. Similarly, meso‐level factors, including national regulations and financial structures, have been previously recognised as critical for PSW implementation (Mirbahaeddin and Chreim 2022). In our evaluation, some of these factors emerged as barriers, particularly the inadequate health insurance billing system for PS services in CMHN. The similarities to international literature in this new context emphasise the broader applicability of existing best practices and guidelines (Repper 2019) for future PSW integration across diverse settings. These factors proved to be more relevant in our evaluation than the length of time PSWs had been part of the CMHN team.

A conducive work culture and opportunities to build relationships between PSWs and non‐PSW staff play a crucial role in facilitating PS. The work culture across the three CMHN teams varied significantly, which profoundly influenced how accepted and well‐integrated the PSWs felt in their roles. Integration was notably hindered where the PSWs perceived the culture as hierarchical, with limited knowledge of recovery and experienced inadequate communication as well as stigmatisation. This phenomenon has been previously described in the literature, where an organisational culture with clear goals, recovery orientation, reflective practice and openness to change was identified as a key factor for successful PSW integration (Ibrahim et al. 2020). Additionally, PSWs must be recognised as a full part of CMHN teams rather than an external addition. Including PSWs in all staff activities and processes has been shown to facilitate the integration process, encouraging non‐peer team members to see PS as a professional role and build relationships as team members (Byrne et al. 2022; de Beer et al. 2023; Reeves et al. 2024). The external counselling and supervision for the PSWs provided by INGA, though seen as helpful during the initial phase of implementation, contributed to the perception that the responsibility for supporting PSWs was more seen as an external matter, rather than a shared duty within the CMHN team. This separation was further reinforced by PSWs' low working hours.

Our evaluation revealed limited knowledge and understanding of PS and recovery principles across all three organisations, with the PSWs' role primarily viewed as providing one‐to‐one client support. This contrasts with the broader understanding that PSWs can contribute at multiple levels, such as supporting professionals and teams in organisational development education and research (Tambuyzer et al. 2014). In CMHN teams, this could potentially encompass contributions to the development of organisational guidelines, training (new) staff, consulting in case conferences and participating in decision‐making processes. Expanding PSW roles beyond direct PSW‐client interaction is essential to ensure recovery‐oriented and participative mental health services (Burr et al. 2022; Slade et al. 2014). The lack of understanding was also evident in the expectation that PSWs acquire specialised psychiatric knowledge, while there was no reciprocal expectation for non‐PSW staff to deepen their understanding of lived experience‐based knowledge, indicating an unequal valuation of knowledge. It is known that the transformative potential of PS is undermined when PSWs feel compelled to adopt medical terminology, as language plays a crucial role in distinguishing PS from traditional psychiatric care (Repper and Carter 2011). This knowledge gap can partly be attributed to inconsistencies in team training, which focused on information transfer rather than mutual learning and reflection. The fact that the decision on whether training was needed was left to the team further contributed to this.

Training and preparation of both PSWs and health professionals are crucial in the successful implementation of PS (Cooper et al. 2024; Mutschler et al. 2022). For health professionals, pre‐implementation and ongoing training are key factors in fostering staff buy‐in and reducing potential stigma toward PSWs (Mutschler et al. 2022). The inconsistent team trainings across the CMHN teams may have led to insufficient preparation. Similarly, variations in training levels were observed among PSWs. Of the three PSWs in our study, only one had completed the EX‐IN training, which is widely regarded as the standard in Switzerland. Previous research has demonstrated that EX‐IN training has positive effects on participants' recovery, stigma resistance and introspection (Hegedüs et al. 2021) and can enhance PSWs' confidence in their role (Hegedüs et al. 2016). Therefore, EX‐IN training can be recommended as a preparatory measure for PSWs. However, unlike in the United States—where a 75‐h certification program enables Certified Peer specialists to provide Medicaid‐reimbursable PS services (Adams 2020)—EX‐IN training in Switzerland is not recognised by health insurance providers and is therefore not obligatory. The lack of financial recognition, highlighted as a key challenge for sustainable PS service development (Burr et al. 2022), could be addressed through official recognition of EX‐IN training or other PSW qualifications for health insurance reimbursement, promoting professionalisation and sustainability.

One of the main limitations of this study is the small sample size, as only three organisations were included, which limits the transferability of the findings. However, the study has several important strengths. First, service user involvement was ensured throughout the data collection and analysis process. Second, the study incorporated perspectives from different professionals and management, as well as client feedback, providing a comprehensive overview. Third, the inclusion of organisations at different stages of PSW implementation allowed the study to explore whether the length of PSWs' involvement influenced their integration within teams. Additionally, the strong focus on quantitative approaches in PS research has been increasingly criticised, with Gillard (2019) emphasising the value of qualitative research, like this study, to capture the complexity and contextual nuances of PS. Our research team's extensive knowledge and expertise in mental health PS and recovery likely influenced the research, as it allowed us to apply these perspectives comprehensively when evaluating the implementation of PSW within the organisations.

## 5 Conclusions

Our evaluation shows that existing guidelines should be utilised when implementing PSWs in new organisations and contexts. A key factor for success is the organization's culture and a strong understanding of PS principles of both non‐peer staff and PSWs, which should be facilitated through appropriate training. While tailored external support can address needs in the initial phase, establishing internal support structures as implementation progresses is crucial for PSW team integration. Additionally, addressing payment and billing issues is crucial to ensuring the long‐term success of PSW integration. The findings of this evaluation offer practical guidance for community mental health nurses on how to implement PSW more effectively and thereby strengthen recovery‐oriented care. By identifying relevant barriers and facilitators, the evaluation directly supports the advancement of mental health nursing in community settings.

## Funding

This work was supported by Ebnet‐Stiftung.

## Ethics Statement

The responsible ethics committee concluded that ethical approval was not required for this study (Req‐2023‐01242) as it is not subject to the Human Research Act in Switzerland. All participants received detailed study information, including their right to withdraw at any time. They provided written consent, confirming voluntary participation, and were given a copy of the consent form.

## Conflicts of Interest

The authors declare no conflicts of interest.

## Data Availability

The data that support the findings of this study are not publicly available due to privacy or ethical restrictions.
